# Indocyanine green fluorescence angiography decreases the risk of anastomotic leakage after rectal cancer surgery: a systematic review and meta-analysis

**DOI:** 10.3389/fmed.2023.1157389

**Published:** 2023-05-12

**Authors:** Shijun Xia, Wenjiang Wu, Lidan Luo, Lijuan Ma, Linchong Yu, Yue Li

**Affiliations:** ^1^Shenzhen Hospital of Guangzhou University of Chinese Medicine, Shenzhen, China; ^2^Shenzhen Traditional Chinese Medicine Anorectal Hospital, Shenzhen, China

**Keywords:** anastomotic leakage, fluorescence angiography, indocyanine green, meta-analysis, rectal cancer

## Abstract

**Background:**

Anastomotic leakage is a serious complication after rectal cancer resection. Intraoperative use of indocyanine green fluorescence angiography (ICGFA) can help prevent anastomotic leakage, but its use is controversial. We conducted a systematic review and meta-analysis to determine the efficacy of ICGFA in reducing anastomotic leakage.

**Methods:**

Relevant data and research published until September 30, 2022, was retrieved from the PubMed, Embase, and Cochrane Library databases, and the difference in the incidence of anastomotic leakage after rectal cancer resection between ICGFA and standard treatment was compared.

**Results:**

This meta-analysis included 22 studies with a total of 4,738 patients. The results showed that ICGFA use during surgery decreased the incidence of anastomotic leakage after rectal cancer surgery [risk ratio (RR) = 0.46; 95% confidence interval (95% CI), 0.39–0.56; *p* < 0.001]. Simultaneously, in subgroup analyses for different regions, ICGFA was found to be used to reduce the incidence of anastomotic leakage after rectal cancer surgery in Asia (RR = 0.33; 95% CI, 0.23–0.48; *p* < 0.00001) and Europe (RR = 0.38; 95% CI, 0.27–0.53; *p* < 0.00001) but not in North America (RR = 0.72; 95% CI, 0.40–1.29; *p* = 0.27). Regarding different levels of anastomotic leakage, ICGFA reduced the incidence of postoperative type A anastomotic leakage (RR = 0.25; 95% CI, 0.14–0.44; *p* < 0.00001) but did not reduce the incidence of type B (RR = 0.70; 95% CI, 0.38–1.31; *p* = 0.27) and type C (RR = 0.97; 95% CI, 0.51–1.97; *p* = 0.93) anastomotic leakages.

**Conclusion:**

ICGFA has been linked to a reduction in anastomotic leakage after rectal cancer resection. However, multicenter randomized controlled trials with larger sample sizes are required for further validation.

## Introduction

Rectal cancer morbidity and mortality are increasing (1). With the advancement in pathophysiology, current individualized treatments for rectal cancer include endoscopic, local surgical resection, systemic treatment, preoperative radiotherapy, local ablation treatment for metastatic tumors, extensive surgery for local and metastatic diseases, targeted therapy, palliative chemotherapy, and immunotherapy, whereas surgery is the cornerstone of curative intent treatment (1). However, rectal cancer patients may face many complications after resection, one of which is anastomotic leakage.

Despite significant advances in surgical techniques and perioperative management in recent years, the incidence of anastomotic leakage after rectal cancer surgery remains between 5 and 19% (2). The occurrence of anastomotic leakage will result in prolonged hospitalization, increased hospitalization costs, increased local recurrence rate, and shortened survival time (3–5). Studies have shown that the risk factors for anastomotic leakage have been determined by age, male gender, smoking, diabetes, previous radiotherapy and chemotherapy, intraoperative complications, anastomotic tension, and low perfusion (2, 6, 7). Among these, insufficient blood perfusion at the anastomosis plays an important role in the pathogenesis of anastomotic leakage (7).

Indocyanine green fluorescent angiography (ICGFA) is widely used in many surgical fields (8–10), including gastrointestinal surgery. The ICGFA allows the surgeon to visualize the blood supply of the intestinal canal and avoid inadequate perfusion of the anastomosis during colorectal surgery. The doctor injected diluted indocyanine green (ICG) into the venous system and monitored the development signal with a fluorescent laparoscope. When the ICG enters the anastomosis area to be observed, the doctor can dynamically observe the blood supply of the colorectal anastomosis with the fluorescent display. Therefore, intraoperative ICG use for intestinal anastomosis blood supply imaging could theoretically become a predictive test to evaluate anastomotic perfusion, reducing the risk of anastomotic leakage. Some meta-analyses have recently revealed that ICGFA can help reduce the incidence of anastomotic leakage after rectal cancer surgery (11–13). However, most of them are not convincing due to the limited number of good qualities RCT studies included and the small number of patients.

According to recently published research, we updated this meta-analysis to evaluate whether this technique can reduce the anastomotic leakage rate of rectal cancer patients after resection.

## Materials and methods

This systematic review and meta-analysis is based on the Preferred Reporting Items for Systematic Reviews and Meta-Analysis statements (PRISMA) (14). Because this study is a meta-analysis, neither institutional review committee approval nor patient-informed consent was required.

### Study strategy

We conducted a comprehensive and systematic search of electronic databases, including PubMed, Embase, and the Cochrane Library; retrieved relevant research published until September 30, 2022; and identified potential articles using the following keyword combinations: (“indocyanine green” OR “fluorescein angiography” OR “fluorescence imaging” OR “indocyanine green-sulfo-OSu” OR “ICG”) AND (“Rectal Neoplasm” OR “Rectal Tumor” OR “Rectal Cancer” OR “Rectum Cancer”). Simultaneously, we searched the reference list and previous comments in these studies for more comprehensive studies that could be included. Two authors (SJ-X and WJ-W) worked independently on this study.

### Anastomotic leakage definition

Anastomotic leakage was defined as a defect in the anastomotic region where there is communication between the intraluminal and extraluminal chambers. Anastomotic leakage was graded by the International Research Group on Rectal Cancer based on its clinical symptoms. Type A had the mildest clinical symptoms, and type C had the most severe clinical symptoms (15). Type A anastomotic leakage is referred to as subclinical leakage or imaging leakage, whereas type B and C anastomotic leakages are referred to as clinical leakage or significant leakage.

### Inclusion and exclusion criteria

The inclusion criteria of this study were as follows: (a) patients undergoing rectal cancer surgery; (b) the report result compares the incidence of anastomotic leakage between the ICGFA group and the control group; and (c) the study design was a randomized controlled trial (RCT), prospective trial, or retrospective trial.

The exclusion criteria were as follows: (a) the study was published in the form of comments, case reports, and letters; (b) the data were insufficient or could not be obtained from the author for meta-analysis; and (c) all inconsistent articles that were ruled by the third examiner (Y-L).

### Data extraction

The two authors (SJ-X and LD-L) conducted independent research and were selected to extract data at the same time based on the inclusion and exclusion criteria. Each included article provided the following information: first author, publication year, research country, research design, and the number of anastomotic leakages.

### Quality assessment

After reading the full text of each included study, the two authors (LJ-M and LC-Y) independently evaluated the quality of the study using the Newcastle Ottawa Scale (NOS). The NOS includes the following four areas: patient selection quality, exposure determination, group comparability, and patient results. The total NOS score ranges from 0 to 9, with a score ≥ 6 indicating high quality.

### Outcomes

The purpose of this study was to compare the incidence of anastomotic leakages in patients undergoing rectal cancer surgery with and without ICG. The secondary analysis was a subgroup analysis, which included the difference in the incidence of anastomotic leakages between regions and grades.

### Statistical analysis

This study is primarily based on binary data. The risk ratio (RR) is used as the effect measure, with a 95% confidence interval. All statistical tests had a significance level of *p* < 0.05 (double-tailed). *I*^2^ statistics was used to determine heterogeneity, with results ranging from 0 to 100%. When *I*^2^ = 0%, heterogeneity was assumed as not observed; *I*^2^ = 25%, heterogeneity was low; *I*^2^ = 50%, heterogeneity was medium; and *I*^2^ = 75%, heterogeneity was high. When *I*^2^ < 50%, a fixed model effect was used. The random model effect was used for all other cases. This study was conducted using the Review Manager software version 5.3 (Nordic Cochrane Center, Cochrane Collaboration, London, UK).

## Result

**Figure 1:**
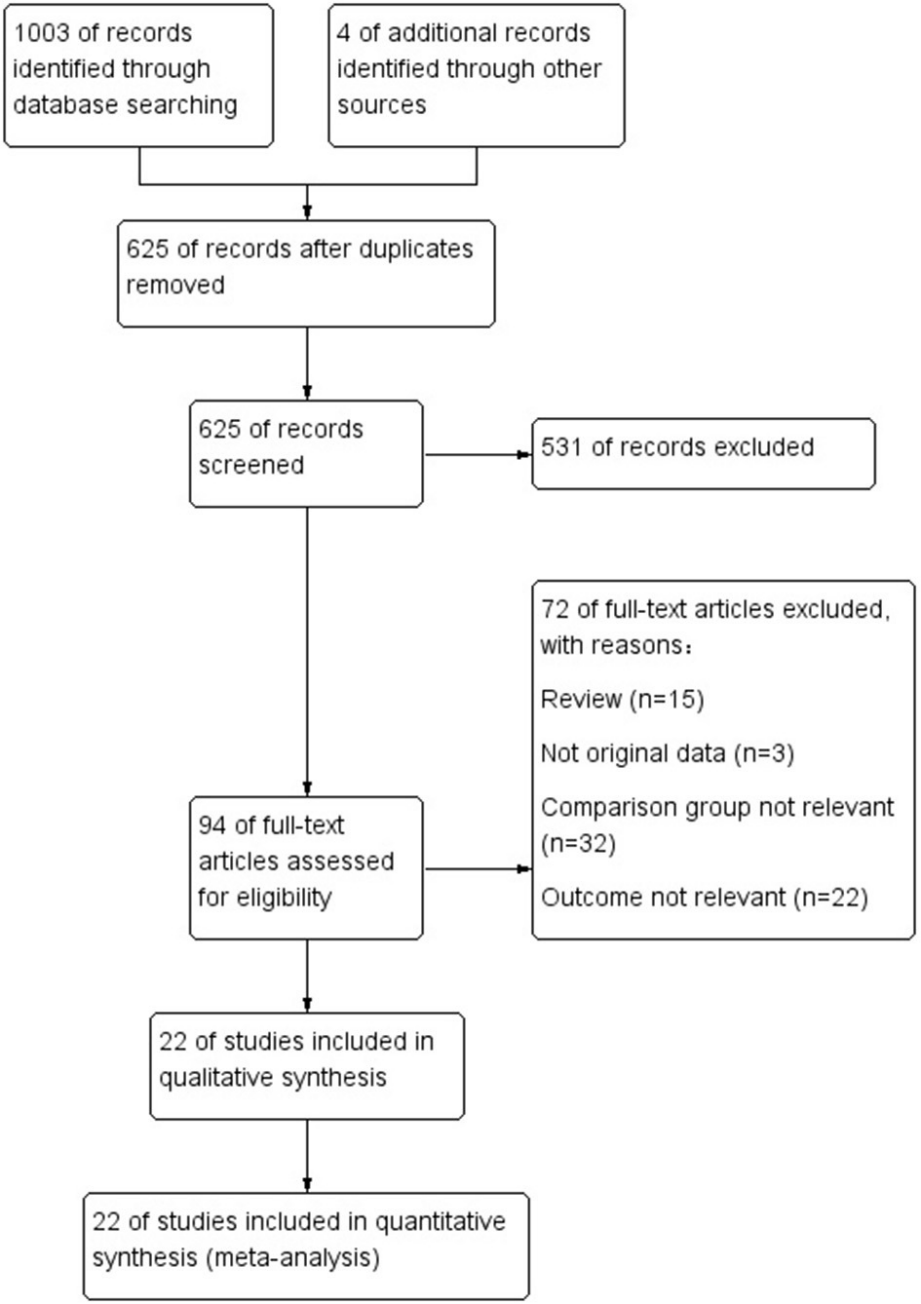
PRISMA flow diagram of study selection.

The detailed process of literature retrieval and screening is shown in Figure 1. A total of 1,007 papers were retrieved. After reading the title and abstract, we excluded 531 studies because they did not meet our inclusion criteria. A total of 22 studies (16–37) met the inclusion criteria among the remaining 94 potential included studies. From 2013 to 2021, 22 studies involving 4,738 patients from 10 countries were published. The sample size of the study ranged from 38 to 657 people. Three RCTs, three prospective studies, and sixteen retrospective studies were conducted. We scored 22 studies from 5 to 9 on the NOS, with 19 (86.4%) rated as high quality. Table 1 shows the basic information included in the study.

**Table 1:** Characteristics of the included trials.

**References**	**Country**	**Study design**	**ICG group**					**Non-ICG group**					**NOS**
			**No**.	**AL no**.	**AL grade A no**.	**AL grade B no**.	**AL grade C no**.	**No**.	**AL no**.	**AL grade A no**.	**AL grade B no**.	**AL grade C no**.	
Jafari et al. (16)	USA	Retrospective	16	1				22	4				6
Kim et al. (17)	Korea	Retrospective	310	2				347	18				8
Boni et al. (18)	Italy	Retrospective	42	0				38	2				7
Brescia et al. (19)	Italy	Retrospective	21	0				31	4				5
Mizrahi et al. (20)	USA	Retrospective	30	0				30	2				8
Ris et al. (21)	Switzerland	Prospectively	90	3				365	39				6
Shapera et al. (22)	USA	Prospectively	58	0				23	1				6
Watanabe et al. (23)	Japan	Retrospective	211	10				211	22				8
De Nardi et al. (24)	Italy	RCT	56	4				53	7				9
Wada et al. (25)	Japan	Retrospective	48	5	0	1	4	101	7	0	4	3	7
Zhou et al. (26)	China	Retrospective	12	1				30	0				6
Alekseev et al. (27)	Russia	RCT	111	16	7	6	3	104	27	19	7	1	9
Bonadio et al. (28)	Italy	Retrospective	33	2	1	1	0	33	7	3	1	3	7
Foo et al. (29)	China	Retrospective	253	9	2	3	4	253	20	5	10	5	6
Hasegawa et al. (30)	Japan	Retrospective	141	4				403	87				8
Impellizzeri et al. (31)	Italy	Retrospective	37	0				36	2				5
Skrovina et al. (32)	Czech Republic	Retrospective	50	5	2	2	1	20	9	8	1	0	6
Wojcik et al. (33)	France	Prospectively	26	1				42	6				7
Ishii et al. (34)	Japan	Retrospective	116	4				104	11				8
Benčurik et al. (35)	Czech Republic	Retrospective	100	9	2	4	3	100	19	13	1	5	7
Jafari et al. (36)	USA	RCT	178	16				169	16				9
Otero Piñeiro et al. (37)	Spain	Retrospective	80	2				204	23				5

ICG, Indocyanine green; AL, anastomotic leakage; NOS, Newcastle Ottawa Scale.

**Figure 2:**
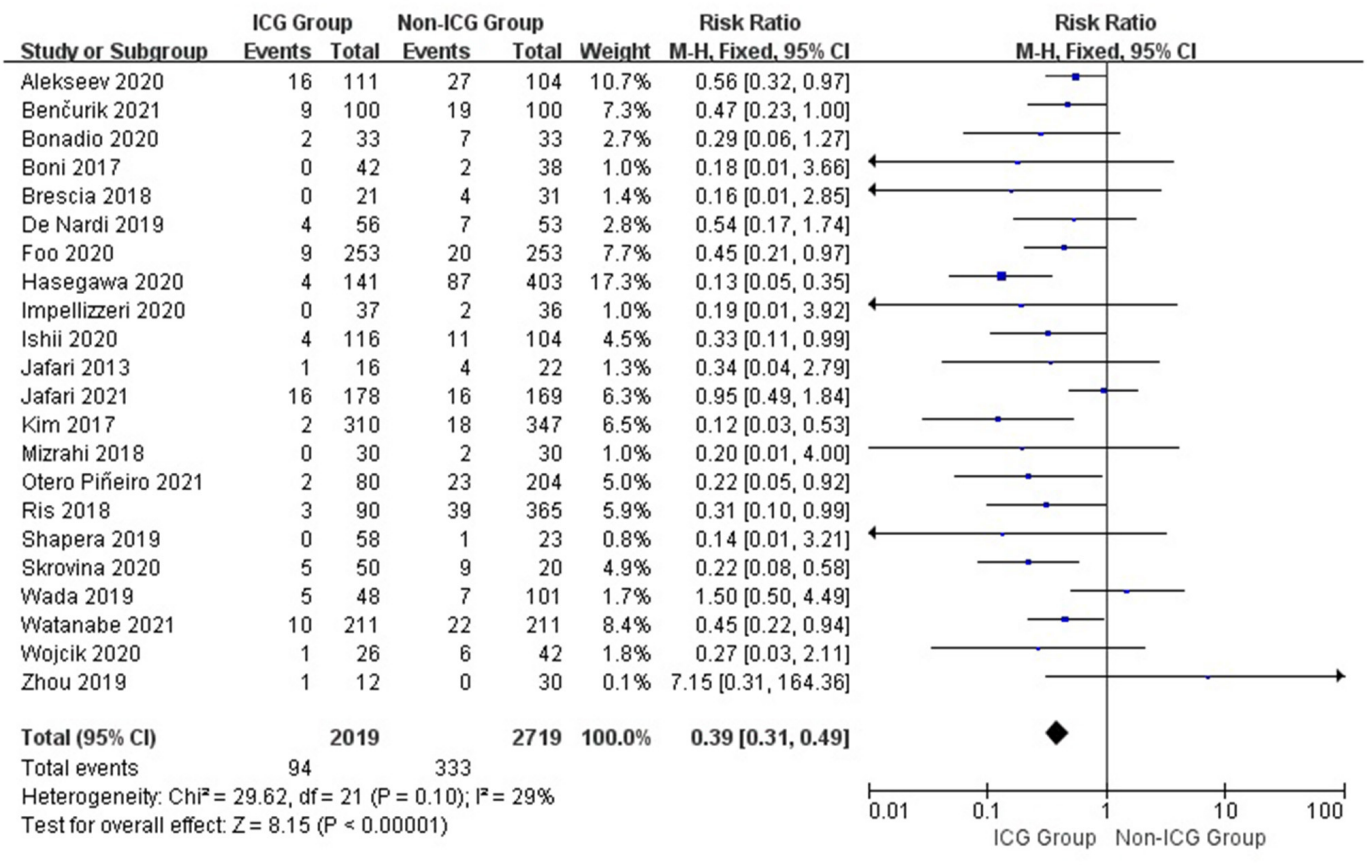
Forest plot of anastomotic leak occurrence in the ICG vs. non-ICG groups.

The combined results of 22 studies revealed that the total anastomotic leakage rates of the ICG and non-ICG groups were 3.7 and 7.6%, respectively (RR = 0.46; 95% CI, 0.39–0.56; *p* < 0.001; Figure 2).

**Figure 3:**
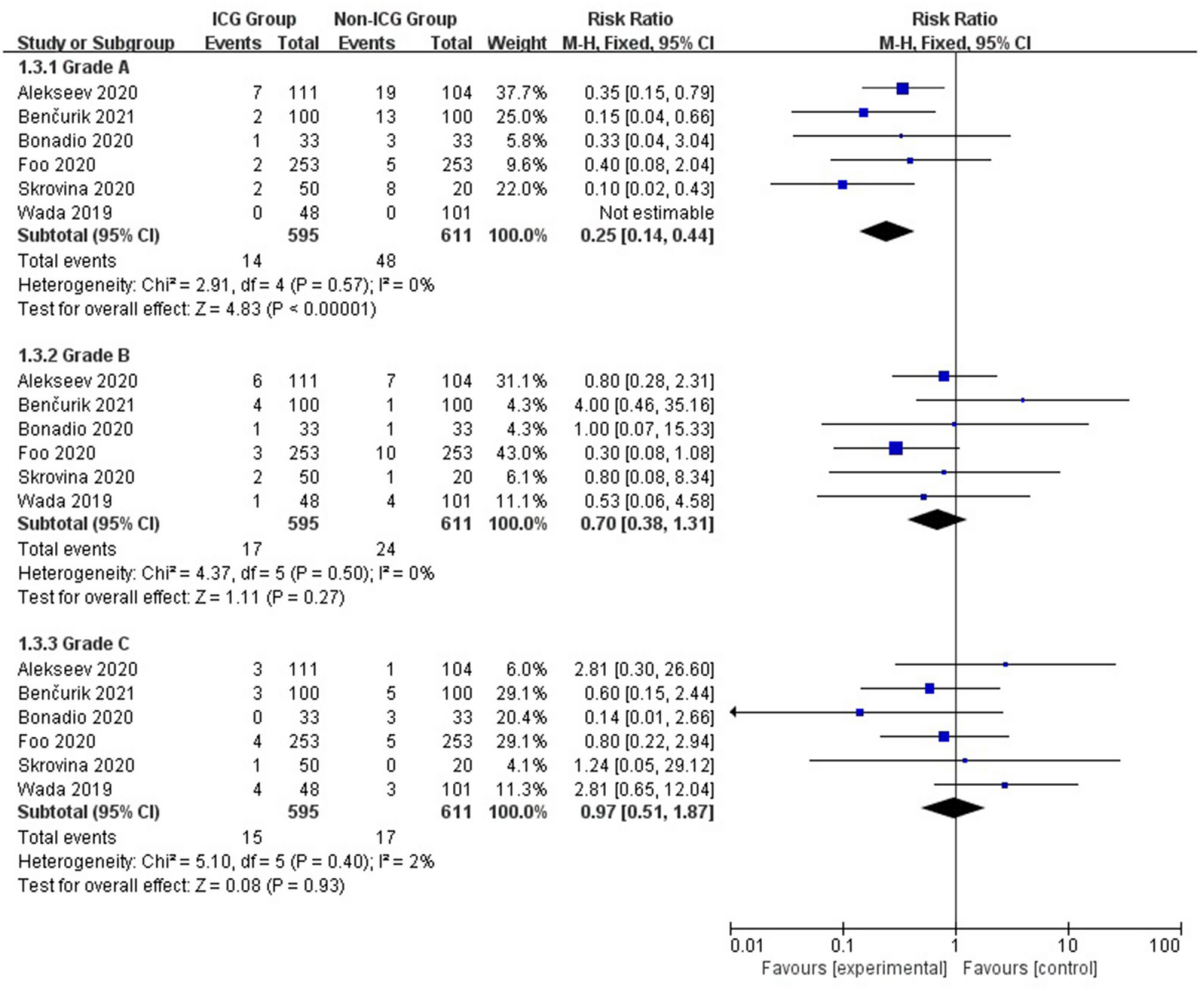
Forest plot of anastomotic leak grade occurrence in the ICG vs. non-ICG groups with sub-analysis.

As in the summary analysis, a similar relationship was observed for grade A anastomotic leakages but not for grades B and C. Grade A anastomotic leakage rate was 2.4% in the ICG group and 7.9% in the non-ICG group (RR = 0.25; 95% CI, 0.14–0.44; *p* < 0.00001); grade B: 2.9 and 3.9%, respectively (RR = 0.70; 95% CI, 0.38–1.31; *p* = 0.27); and grade C: 2.5 and 2.8%, respectively (RR = 0.97; 95% CI, 0.51–1.97; *p* = 0.93; Figure 3).

**Figure 4:**
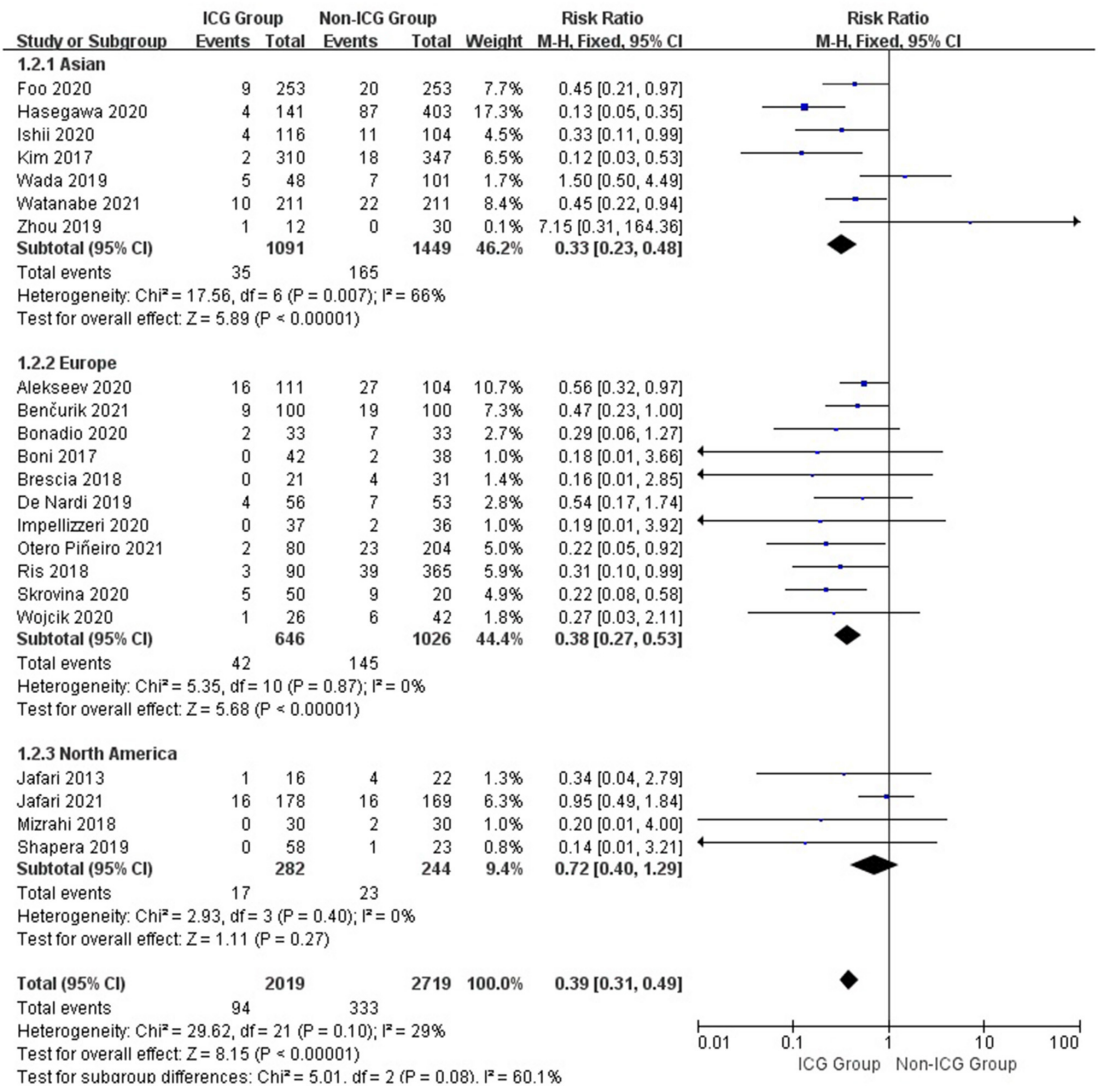
Forest plot of anastomotic leakage grade occurrence with different regions in the ICG vs. non-ICG groups with sub-analysis.

The subgroup analysis of 22 studies from different regions revealed that the rate of anastomotic leakage in the ICG group was 3.2% in Asia compared with 11.4% in the non-ICG group (RR = 0.33; 95% CI, 0.23–0.48; *p* < 0.00001), 6.5 and 14.1% in Europe (RR = 0.38; 95% CI, 0.27–0.53; *p* < 0.00001), and 6.0 and 9.4% in North America (RR = 0.72; 95% CI, 0.40–1.29; *p* = 0.27; Figure 4).

### Publication bias

**Figure 5:**
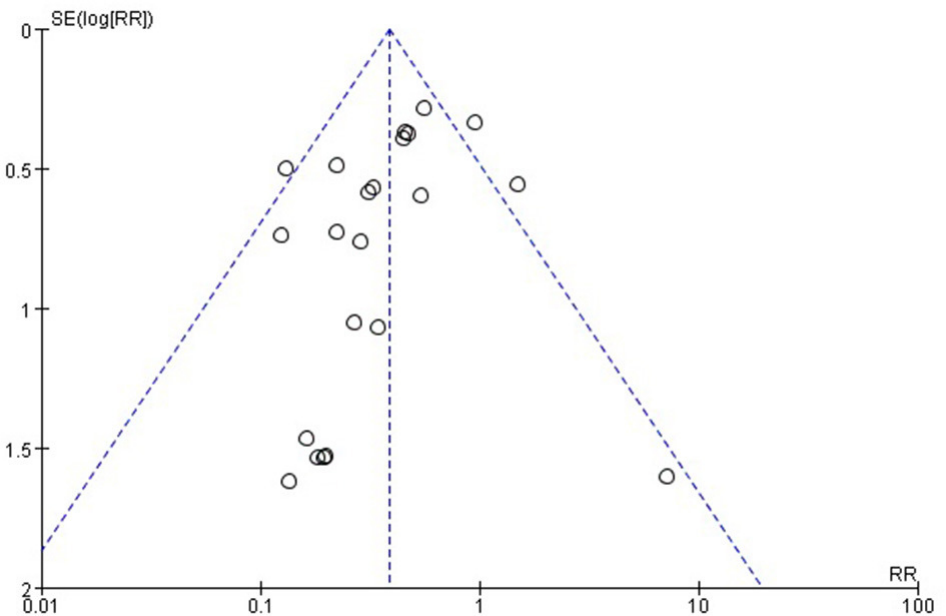
Funnel plot diagram.

Funnel plots were used to assess potential publication bias in a meta-analysis of the relationship between ICG use and anastomotic leakages. The funnel plot is symmetrical, as shown in Figure 5, indicating that the risk of deviation published in this study is low.

## Discussion

The largest meta-analysis on the impact of ICGFA on anastomotic leakage in patients with rectal cancer surgery included 22 articles and 4,738 patients. The results of a pooled analysis of these patients revealed that the use of ICGFA during surgery was associated with a lower incidence of anastomotic leakage after rectal cancer resection. The results of a subgroup analysis of these patients revealed that the use of ICGFA during surgery in Asia and Europe was associated with a lower incidence of anastomotic leakage after rectal cancer resection and the use of ICGFA during surgery was associated with a lower incidence of grade A anastomotic leakage after rectal cancer resection.

The negative impact of anastomotic leakage after rectal cancer surgery cannot be ignored. Anastomotic leakage will result in prolonged hospitalization, increased hospitalization costs, increased local recurrence rate, and shortened survival time (38). Many studies have determined that anastomotic leakage is an independent risk factor for low long-term survival rate of patients with rectal cancer (39, 40). The study shows that anastomotic leakage risk factors have been determined in many situations, such as age, male gender, smoking, diabetes, previous radiotherapy and chemotherapy, intraoperative complications, anastomotic tension, and low perfusion. These risk factors are partly related to patients, tumors, and surgery. Some risk factors are objective and cannot be changed, whereas other risk factors can be changed. Anastomotic perfusion is one of the few variables that can be changed.

ICG is a biocompatible near-infrared contrast agent that can be excited by external light with a wavelength of 750–800 nm and emit near-infrared light with a longer wavelength, allowing tissues and organs to develop. Because Nagata first used ICG in colorectal surgery in 2006, this technology has demonstrated significant research value and promising application prospects in the auxiliary diagnosis and treatment of colorectal cancer (41, 42). It has been reported in the literature that ICG near-infrared imaging technology can improve the visualization of tumor lesions, improve the detection rate of lymph nodes, and reduce the incidence of anastomotic leakage in laparoscopic colorectal cancer surgery (42).

The ICG near-infrared imaging technique is used to evaluate the intestinal blood supply at the anastomotic site during colorectal cancer surgery. Before the proximal intestinal wall is disconnected, the operator can use clinical judgment to select the resection line under white light or visible light and mark the “pre-resection line” on the intestinal wall without damage. However, it should be noted that unipolar or bipolar electrical equipment should be used to burn the mark to avoid damaging the local blood supply of the intestinal wall and ultimately affecting the accuracy of the assessment. After the location of intestinal resection was determined, ICG was injected intravenously for the first time, and vascular perfusion was monitored using a near-infrared camera system. If the ICG vascular perfusion imaging is good within 60 s, the intestinal blood supply is likely to be adequate. ICG has a median development time of 35 (29–44) s and a duration of 3 min. Record the boundary between perfusion and non-perfusion tissues of the intestinal tube and compare it with the previously marked “pre-resection line” of the intestinal tube before cutting the intestinal wall along the ischemic line. If the blood perfusion at the “pre-resection line” of the intestinal tube is deemed insufficient, the “pre-resection line” at the proximal end of the intestinal tube should be moved to a position with adequate blood perfusion. ICG was injected intravenously again after intestinal anastomosis. The perfusion after anastomosis was evaluated using a fluorescence system, and the blood supply and appearance of the intestinal wall were observed to determine whether the surgical strategy should be changed and intestinal anastomosis performed again.

The use of ICGFA in assessing anastomotic perfusion has been concerned in recent years due to its relative ease of use, low cost, and satisfactory safety (17, 21). Recently published meta-analyses (11–13) showed that the risk of anastomotic leakage after rectal surgery in patients with ICGFA was significantly reduced, which was consistent with our results. However, due to the limited number of articles included, the majority of the studies are inconclusive. In comparison to these studies, the advantage of our meta-analysis lies in the inclusion of all relevant RCT, prospective, and retrospective studies, allowing us to analyze a larger sample size than previous meta-analyses and produce more convincing research results. Simultaneously, our research object is more specific and singular, which is patients with rectal cancer undergoing surgical treatment. In patients undergoing rectal cancer resection, the incidence of anastomotic leakage is higher than in patients undergoing colon cancer surgery. Therefore, this group is most likely to benefit from this intervention.

According to a summary analysis of all patients, the use of ICGFA during surgery is associated with a reduction of anastomotic leakage after rectal cancer resection (RR = 0.25; 95% CI, 0.14–0.44; *p* < 0.00001). We found that using ICGFA during surgery was associated with a reduction in the incidence of anastomotic leakage after rectal cancer resection in Asia and Europe (RR = 0.33; 95% CI, 0.23–0.48; *p* < 0.00001 and RR = 0.38; 95% CI, 0.27–0.53; *p* < 0.00001) but not in North America (RR = 0.72; 95% CI, 0.40–1.29; *p* = 0.27). We believe that this is due to fewer studies and a smaller sample size, which causes heterogeneity in the analysis of results. The use of ICGFA during surgery is associated with a reduction in the incidence of grade A anastomotic leakage after rectal cancer resection (RR = 0.25; 95% CI, 0.14–0.44; *p* < 0.00001), but there is no difference in the incidence of grades B and C anastomotic leakages (RR = 0.70; 95% CI, 0.38–1.31; *p* = 0.27 and RR = 0.97; 95% CI, 0.51–1.97; *p* = 0.93). Therefore, we can conclude that ICGFA cannot reduce the occurrence of type B and C anastomotic leakages after rectal cancer resection. Because both the ICGFA intervention and control groups had a low incidence of types B and C anastomotic leakage, ICGFA could not reduce the incidence of types B and C anastomotic leakage after rectal cancer resection.

At the same time, we recognize that this study has some limitations. First, the diagnosis of anastomotic leakage varied across the studies. Some studies were diagnosed solely through radiology, whereas others were diagnosed through physical examination and endoscopy as well. Second, the dose of ICG administered and timing of the included studies were inconsistent. Third, restricted by the articles included, this study cannot be analyzed according to the level of rectal cancer. Finally, the number of RCTs in the study is insufficient, which may increase the risk of deviation. These limitations may result in heterogeneity in the analysis of research findings.

## Conclusion

In conclusion, the results of this systematic review and meta-analysis show that using ICGFA during rectal cancer surgery is associated with a significant reduction in AL. However, given the limitations mentioned above, more multicenter RCT studies with a large sample size are required in the future to demonstrate our research findings.

## Data availability statement

The datasets presented in this study can be found in online repositories. The names of the repository/repositories and accession number(s) can be found below: https://pubmed.ncbi.nlm.nih.gov/.

## Author contributions

SX: analysis, interpretation of data, and drafting the article or revising it critically for important intellectual content. WW: conception and design and final approval of the version to be published. LL, LM, LY, and YL: acquisition of data. All authors contributed to the article and approved the submitted version.
